# Large-Area and Ultrathin MEMS Mirror Using Silicon Micro Rim

**DOI:** 10.3390/mi12070754

**Published:** 2021-06-26

**Authors:** Myeong-Su Ahn, Jaehun Jeon, Kyung-Won Jang, Ki-Hun Jeong

**Affiliations:** Department of Bio and Brain Engineering, Korea Advanced Institute of Science and Technology (KAIST), 291 Daehak-ro, Yuseong-gu, Daejeon 34141, Korea; m.s.ahn@kaist.ac.kr (M.-S.A.); wjswogns1206@kaist.ac.kr (J.J.); kwjang@kaist.ac.kr (K.-W.J.)

**Keywords:** large-area and ultrathin MEMS mirror, bimorph thermal actuator, statically actuated MEMS mirror, silicon rim microstructures

## Abstract

A large-area and ultrathin MEMS (microelectromechanical system) mirror can provide efficient light-coupling, a large scanning area, and high energy efficiency for actuation. However, the ultrathin mirror is significantly vulnerable to diverse film deformation due to residual thin film stresses, so that high flatness of the mirror is hardly achieved. Here, we report a MEMS mirror of large-area and ultrathin membrane with high flatness by using the silicon rim microstructure (SRM). The ultrathin MEMS mirror with SRM (SRM-mirror) consists of aluminum (Al) deposited silicon nitride membrane, bimorph actuator, and the SRM. The SRM is simply fabricated underneath the silicon nitride membrane, and thus effectively inhibits the tensile stress relaxation of the membrane. As a result, the membrane has high flatness of 10.6 m^−1^ film curvature at minimum without any deformation. The electrothermal actuation of the SRM-mirror shows large tilting angles from 15° to −45° depending on the applied DC voltage of 0~4 V_DC_, preserving high flatness of the tilting membrane. This stable and statically actuated SRM-mirror spurs diverse micro-optic applications such as optical sensing, beam alignment, or optical switching.

## 1. Introduction

Microelectromechanical system (MEMS) mirrors allow for diverse applications [1] such as microscopy [2], FTIR (Fourier Transform Infrared) spectroscopy [3], optical switch [4], display [5,6], and structured illumination [7]. They obviously require a large-area and flat mirror for efficient light-coupling and high spatial resolution. The large-area mirror often overcomes the film deformation by employing a thick mirror membrane, and thus maintains the high flatness of the mirror during the actuation. For instance, a single-crystal-silicon (i.e., SCS) as thick as a few tens of micrometers is commonly used as a flat substrate for the movable membrane due to its low residual stress and low surface roughness [8,9,10]. However, the thick membrane significantly limits an actuation displacement without high enough driving powers due to its non-negligible mass, and thus hampers the MEMS actuator with further large-area mirrors. In addition, a thin actuator is often desirable for a wide range of actuation [11], but monolithic fabrication of a thick mirror membrane and a thin actuator is challenging.

A thin membrane MEMS mirror provides several advantages such as low cost, low voltage operation, and high compatibility for large-area configuration due to its low weight [11,12]. In particular, an electrothermal actuator incorporating a thin membrane as well as thin bimorph structures is highly favorable for fast heat dissipation [13,14] and large actuation ranges [15]. However, the freely moving thin membrane is significantly vulnerable to diverse film deformation such as cracks [16,17] and buckling [18,19], as the membrane area is further increased. Film deformation occurs when the released thin membrane tends to shrink or expand due to its residual tensile or compressive stress [20]. Residual stresses often result from atomic rearrangement (i.e., dissociation of initial bond and rearrangement to stable bond) during and after the film growth process (i.e., chemical vapor deposition; CVD) [21]. For instance, a stoichiometric silicon nitride (Si_3_N_4_) thin film of 0.2–0.3 μm has a large tensile residual stress of 1100–1300 MPa [22,23], caused by dissociation of Si–H and N–H bonds, and rearrangement of the dangling bonds to stable Si–N bonds [21]. Some technical efforts have been made to reduce or compensate the stresses either by manipulating gas ratio during the film growth [24], or by employing the sandwiched [25] or perforated structures [18], but it is still challenging to facilitate a freely moving thin membrane with a large area and high flatness. Here, we report a large area and ultrathin MEMS mirror with high flatness by using a silicon rim microstructure (SRM). The SRM effectively inhibits the tensile stress relaxation of the large area silicon nitride membrane of 5 mm (w) × 4.5 mm (l) × 1 μm (h). As a result, the ultrathin MEMS mirror with SRM (SRM-mirror) exhibits a high enough film flatness of 10 cm radius of curvature without noticeable film deformation.

## 2. Design and Parameters of SRM-Mirror

### 2.1. SRM-Mirror

**Figure 1:**
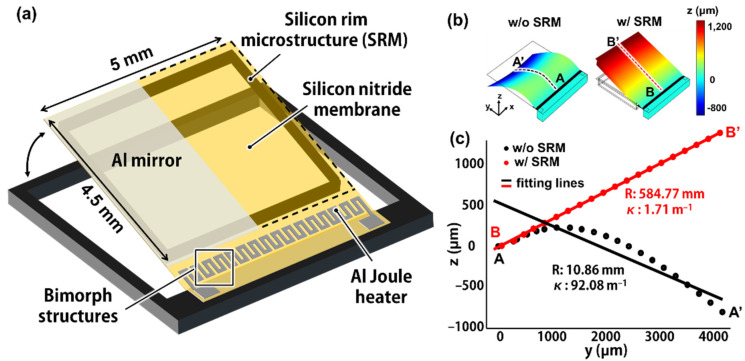
(**a**) Schematic of a silicon micro (μ) rim-loaded MEMS mirror (SRM-mirror), consisting of aluminum (Al) thin mirror, silicon nitride membrane, bimorph structures, and the silicon micro rim; (**b**) Numerical analysis on film flatness of SRM-mirror depending on the rim, by using finite element method (FEM) simulation; (**c**) The cross-sectional profiles (i.e., A-A′, B-B′ in (**b**)) of the thin mirror membrane with/without the SRM, and the calculated curvature (κ) and radii of curvature (R).

The SRM-mirror consists of an ultrathin silicon nitride membrane, aluminum (Al) thin mirror, bimorph structures, and the SRM (Figure 1a). The bimorph structures comprise an Al Joule heater and silicon nitride membrane, which are highly suitable for large stroke due to the large difference in coefficient of thermal expansion (CTE) (see the Table 1 for the detailed variables). The Al Joule heater and the Al mirror are defined on the same silicon nitride membrane, resulting in a high fill factor of mirror area as well as facile fabrication. The SRM is simply attached as a rectangular frame underneath the silicon nitride membrane to prevent bending or other film deformation. The finite element method (FEM; COMSOL Multiphysics^®^) was used to calculate an initial state (i.e., no electric potential applied) of the thin MEMS mirror with and without the SRM (Figure 1b). The residual stresses of silicon nitride membrane and Al Joule heater were assumed to be uniform in x and y direction (σ_xx_, σ_yy_). The membrane without SRM shows substantial deformation such as vertical bending and lateral warping due to the residual stress of silicon nitride thin film and Al Joule heater. In contrast, the SRM-mirror achieves high flatness without any significant deformation. The flatness of the mirror membrane was quantitatively analyzed by calculating the cross-sectional displacements along the vertical cut line (i.e., dotted line A-A′, B-B′ in Figure 1b), and the radius of curvature (Figure 1c). The SRM-mirror shows high flatness of about 60 cm radius of curvature (ROC), and curvature (i.e., κ; inverse of ROC) of 1.71 m^−1^, which is about 50 times lower than the curvature of the SRM-free thin membrane.

### 2.2. Design Parameters

**Figure 2:**
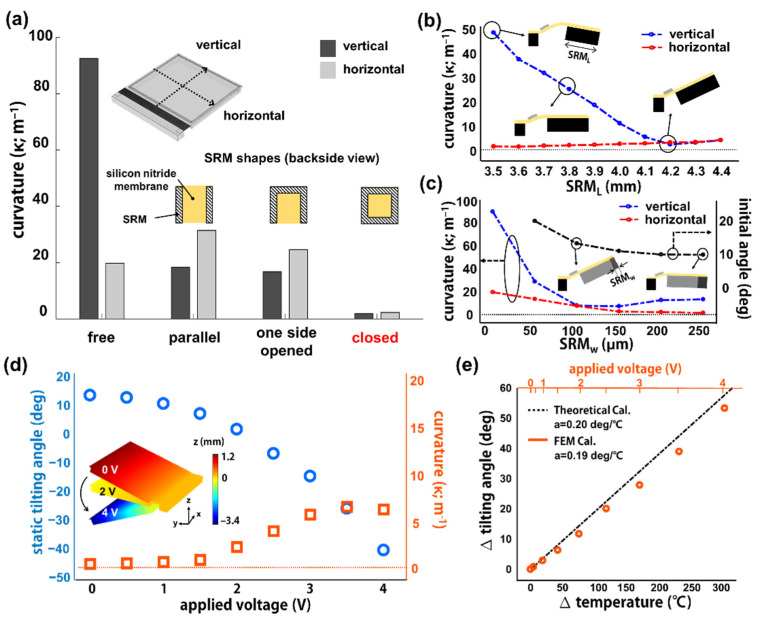
Numerical analysis on the film flatness and electrothermal actuation of SRM-mirror. Calculated curvatures of SRM-mirror depending on (**a**) the shape; (**b**) length; (**c**) width of silicon rims; (**d**) Tilting angles and film curvatures of SRM-mirror during electrothermal actuation with voltages increasing from 0 V_DC_ to 4 V_DC_; (**e**) Comparison of FEM calculation with theoretical calculation.

Various design parameters of SRM-mirror including shapes, lengths, and widths of the rim were investigated to achieve a high degree of film flatness. Figure 2a shows the curvatures of silicon nitride membrane depending on the shape of silicon rims such as parallel, one side-opened, and closed shapes. The vertical and horizontal curvatures were averaged from the individual curvatures along the five lines for the vertical direction and five lines for the horizontal direction of the membrane, respectively (Figure S1). The vertical curvature is significantly decreased as the silicon rims of parallel shape or one side-opened shape are attached to the membrane. The large vertical curvature of the silicon rim-free membrane results from the thin film bending along the vertical direction, which is effectively inhibited by the silicon rim attached along the vertical direction. The horizontal curvature is slightly increased as the silicon rims with parallel shapes are attached. This is because the tensile stress of the membrane is relaxed through the rim-free space, resulting in the film shrinkage along the horizontal direction and thus the lateral warping occurs. The silicon rim with a one side-opened shape slightly improves the horizontal flatness due to an additional single rim along the horizontal direction, but lateral warping still occurs through the rim-free space. In contrast, the closed silicon rim effectively inhibits the tensile stress relaxation of the large area membrane, exhibiting the lowest vertical and horizontal curvatures without vertical bending or lateral warping.

Figure 2b shows the curvatures of the membrane with the closed silicon rim, depending on the SRM length (SRM_L_). The SRM length is defined as the length of the silicon rim along the vertical direction, as shown in inset figures. The lengths of the horizontal silicon rims are constant and thus the horizontal curvatures show no significant changes. In contrast, the vertical curvature clearly decreases as the SRM length increases. It turns out that the stress relaxation through the whole membrane is effectively inhibited as the freestanding area closed by SRM becomes large. The vertical and horizontal curvatures slightly increase with further increased SRM length, because the stress is partially relaxed through the further widen freestanding area closed by SRM. As a result, the SRM-mirror shows a high flatness of 2 m^−1^ curvature when the SRM is long enough to inhibit stress relaxation through the whole freestanding membrane.

The widths of silicon rim (SRM_w_) were also investigated for their vertical and horizontal curvatures (Figure 2c). Both the curvatures dramatically decreased as the rim widths increased from 0 μm (i.e., no rim) to 50 μm. The vertical curvature then slightly increased as the rim widths further increased due to gravitational downwards bending, which is proportional to the rim widths. In addition, the initial tilting angles of SRM-mirror decreased depending on the SRM_w_, which is highly related to the downward bending of the freestanding membrane. Figure 2d shows the calculated tilting angles and the corresponding curvature variations depending on applied DC voltage during electrothermal actuation. The simulation of electrothermal actuation was performed by incorporating three physics engines such as “solid mechanics”, “heat transfer in solids”, and “electric currents” in the stationary mode. The tilting angles of SRM-mirror vary from +13° to −40° when the applied voltages are increased from 0 V_DC_ to 4 V_DC_. The membrane curvature is slightly increased from 1.4 m^−1^ to 6.7 m^−1^ during actuation, which is still noticeably lower than the curvature of the SRM-free membrane of 92.1 m^−1^. The inset figure describes the calculated electrothermal actuation of the SRM-mirror, preserving high film flatness. Furthermore, the FEM-calculated electrothermal actuation of SRM-mirror was confirmed by theoretical calculation for bimorph structures (Figure 2e). The tilting angles and the corresponding temperature variances (∆T) of the bimorph structures were calculated using FEM and a theoretical equation [15], which shows highly coherent linear lines with slopes of 0.19 and 0.20 deg/°C, respectively.

## 3. Microfabrication of SRM-Mirror

**Figure 3:**
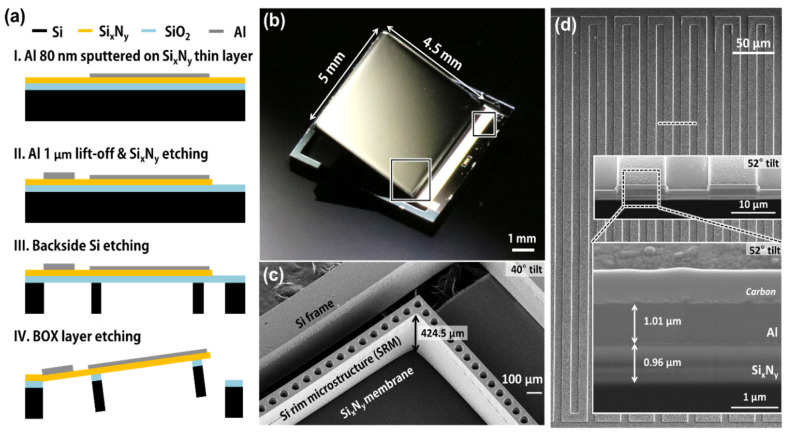
Microfabrication of SRM-mirror. (**a**) Microfabrication procedures including (I) Al mirror sputtering, (II) lift-off of Al Joule heater, (III) backside DRIE of silicon, and (IV) isotropic etching of thermal oxide layer; (**b**) Optical image of the SRM-mirror; (**c**) Tilted SEM image of SRM-mirror and the attached SRM (backside view); (**d**) SEM image of Al Joule heater. Insets depict a cross-sectional view of bimorph structures comprising of the Al Joule heater and silicon nitride membrane.

The SRM-mirror was simply fabricated using Al evaporation and backside silicon DRIE (Deep Reactive Ion Etching) (Figure 3a). The working substrate was prepared using a low-stress silicon nitride (Si_x_N_y_) layer, a thermal oxide layer, and a silicon substrate. A 1.5 μm thick oxide layer was thermally grown (TERA300SE by Wonik IPS) on a 6-inch silicon wafer as an etch-stop layer during the silicon DRIE. A 1 μm thick low-stress silicon nitride layer was then deposited (PF-D82 by P&Tech, Cheongju-city, Republic of Korea) on the thermal oxide layer using LPCVD (Low Pressure Chemical Vapor Deposition) by manipulating the gas ratio of ammonia (NH_3_) to dichlorosilane (SiH_2_Cl_2_; DCS) as 40: 200 sccm. An 80 nm thick Al thin film was sputtered and then the 1 μm thick Al line patterns for Joule heater were defined by using the lift-off process. The silicon nitride layer was etched to define a freestanding membrane using RIE (Reactive Ion Etch). The backside silicon DRIE was finally carried out after PR (Photo Resists) patterning of the SRM. Figure 3b shows the optical image of the fabricated SRM-mirror with the large and flat freestanding membrane of 5 mm × 4.5 mm. Underneath the freestanding membrane, the SRM is well attached with 120 μm width and 425 μm height as same as the thickness of silicon substrate (Figure 3c). The SRM also has micro-hole arrays with 30 μm radius and 90 μm pitch to reduce gravitational side-effects. Figure 3d shows an SEM image of the Al Joule heater on the silicon nitride membrane. The Al Joule heater has continuous Al micro-wires with 11 μm width and 20 μm pitch. The inset SEM images describe the cross-sectional view (52° tilt) of bimorph structures consisting of Al wires and silicon nitride membrane, which are about 1 μm thick each.

## 4. Mechanical and Optical Characterization of SRM-Mirror

**Figure 4:**
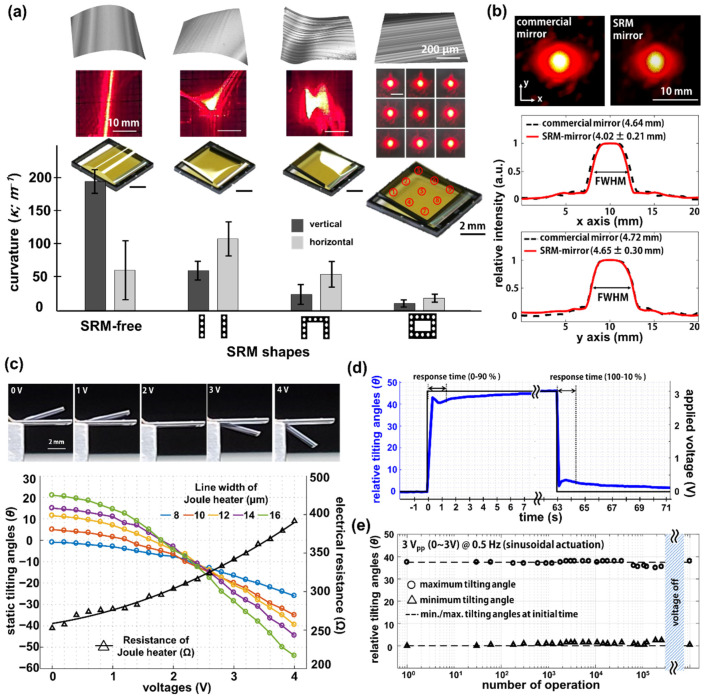
Mechanical and optical characterization of the SRM-mirror. (**a**) Measured film curvatures of the SRM-mirror depending on the shape of silicon rims. Insets correspond to 3D-reconstructed images of membrane (top row), images of reflected laser spots (middle row), and optical images (bottom row); (**b**) Beam profile analysis of reflected laser beam spots from a commercial mirror and SRM-mirror; (**c**) Measured tilting angles of the SRM-mirror and resistances of the Al Joule heater during electrothermal actuation with varied voltages from 0 V_DC_ to 4 V_DC_. Insets are side-view images of the SRM-mirror during actuation; (**d**) Step response of the SRM-mirror with static actuation between the voltages of 0 V_DC_ and 3 V_DC_; (**e**) Fatigue test of the SRM-mirror. The sinusoidal waveform with 0.5 Hz/3 V_pp_ (0–3 V) was applied for three days. The minimum and maximum tilting angles were measured at the specified time.

The SRM-mirror was mechanically and optically characterized to measure the membrane flatness and tilting angles by electrothermal actuation. Figure 4a shows the optically measured film deformation and the curvatures calculated from the measured values, depending on the shape of the SRM. The top row insets in Figure 4a indicate three-dimensional shapes of the freestanding membranes reconstructed from the height profiles, measured by confocal optical profiler (m-surf by Nanofocus AG, Max-Planck-Ring 48, 46049 Oberhausen, Germany). Unlike the membrane with closed SRM, the reconstructed 3D figures show significant film deformation in case of SRM-free, parallel SRM, and one side-opened SRM. The membrane curvatures were calculated from the measured height profiles, and the SRM-mirror with closed SRM shows the lowest curvatures of 10.6 and 18.7 m^−1^ for vertical and horizontal directions, respectively (see Figure S3 for roughness at micron scale). In addition, the curvature variations between different shapes of SRM show a high coherence with the FEM calculation results. The middle row insets describe the on-screen shapes of laser beam spot, reflected from each SRM-mirror. The reflected spots of SRM-mirror with SRM-free, parallel SRM, and one side-opened SRM exhibit severe distortions such as a prolonged line shape, an enlarged triangular, or a bow-tie shape, due to the uneven mirror surfaces resulting from film deformation. Distortion and film deformation were also confirmed by the optical images of SRM-mirrors shown in the bottom row insets. In contrast, the SRM-mirror with closed SRM clearly shows the circular shapes of reflected laser spots in nine different positions on the membrane. The spots from the SRM-mirror were quantitatively compared with laser spots reflected from a commercial mirror (E02; broadband dielectric mirror from Thorlabs, Inc.) by measuring the FWHM (full width half maximum) of each beam profile (Figure 4b and Video S1 from Supplementary Materials). The averaged FWHM of beam profiles from the SRM-mirror are 4.02 mm and 4.65 mm along the *x*-axis and *y*-axis, respectively, which represents an ellipsoidal shape close to a circle. It turns out that the SRM-mirror has a small tilting angle (i.e., slight deviation from the horizontal plane) so that the laser spot on the screen is slightly stretched along the vertical direction. However, the beam profile from the SRM-mirror is still similar with that from the commercial mirror, which indicates the high flatness of the SRM-mirror.

Electrothermal actuation of the SRM-mirror was demonstrated depending on the width of Al wires (Figure 4c). The measured static tilting angles show that the SRM-mirror with a wide Al wire, e.g., 16 μm, exhibits a wide scan range of +21° to −54° as the applied DC voltages increase from 0 V_DC_ to 4 V_DC_. This is because the wide Al wire has a low resistance, and the temperature variance (∆T) of the Al Joule heater is inversely proportional to the resistance at a static voltage. However, the Joule heater with further wide Al wires often suffers from fabrication failures leading to electrical short circuits, due to the high aspect ratio of the sacrificial layer during the lift-off process. Therefore, an SRM-mirror with 14 μm width showing a scan range of 60° was used for further analysis. The electrical resistances of the heater were also measured depending on the applied voltage, which are coherent with theoretical expectations based on Ohm’s law and the resistance of conductive wires. The insets in Figure 4c show the side-view images of the SRM-mirror during the electrothermal actuation. The step response of the SRM-mirror was then investigated to measure the response time for impulse input (Figure 4d). The SRM-mirror was actuated with static voltages between 0 V_DC_ (off) and 3 V_DC_ (on), and the tilting angles of the SRM-mirror were continuously measured by monitoring the reflected laser spot every 100 msec. There was a rise time (0–90%) and fall time (100–10%) for a scan range of 45° are 1.3 s and 1.4 s, respectively. The relatively long response times result from a low damping ratio (ζ; i.e., underdamped) of the large area and ultrathin silicon nitride membrane. The result implies that the SRM-mirror is applicable as a static or low-frequency controlled MEMS mirror, rather than a high-speed scanner (Figure S4 for modal analysis at eigenfrequencies). A mechanical fatigue test of the SRM-mirror was also performed depending on the number of operations by applying sinusoidal AC voltages with 3 V_pp_ (0~3 V) and 0.5 Hz (Figure 4e and Video S2 from Supplementary Materials). The sinusoidal waveform with the low frequency was chosen to prevent abrupt changes at peak voltage (i.e., 3 V), and to provide sufficient cooling time at 0 V. The SRM-mirror was then continuously actuated for three days to investigate functional degradation resulting from long-term heat accumulation. The scanning range shows stable actuation until 4 × 10^4^ operations, which then decreases about 10% after the 10^5^ operations due to heat accumulation in the Al Joule heater and silicon nitride membrane. However, the scanning range fully recovered soon after sufficient cool-down of the SRM-mirror within 10 s, which was also confirmed in the results of the step response. As a result, the mechanical and optical characterization of the SRM-mirror clearly demonstrated not only high flatness of a large area ultrathin membrane, but also a wide scan range during electrothermal actuation.

## 5. Conclusions

To conclude, we have successfully demonstrated a large area and ultrathin MEMS mirror by using the SRM, which is attached underneath a large and thin freestanding membrane of 5 mm × 4.5 mm × 1 μm. The simple fabrication of the SRM using the silicon DRIE allows the SRM-mirror with high flatness of 10.6 m^−1^ curvature without any film deformation. The profile of laser beam spots reflected from the SRM-mirror is highly coherent with that from a commercial mirror. The SRM-mirror also shows a wide scan angle of 60° during electrothermal actuation with applied DC voltages of 0–4 V_DC_, thanks to the thin bimorph structures facilitated by the thin freestanding membrane. The step response and fatigue test of the SRM-mirror show a response time of less than 1.4 s and a fast functional recovery. This large area and statically actuated SRM-mirror provides a new platform for diverse active micro-optic applications such as optical sensing, beam alignment, or optical switching.

## Figures and Tables

**Table 1:** Geometric and mechanical parameters of the SRM-mirror.

Parameters	Values	Remark
**Geometric**		
Si_x_N_y_ membrane area	5 mm (w) × 4.5 mm (l)	
Si_x_N_y_ membrane thickness	1 μm	Figure S2a
SiO_2_ thickness	1.5 μm	
Al mirror thickness	80 nm	Figure S2b
Al heater (line pattern) thickness	1 μm	Figure S2a
Al heater (line pattern) length	500 μm	
Al heater (line pattern) width	14 μm	
Al heater (line pattern) pitch	20 μm	
SRM length (horizontal/vertical)	4.9 mm/4.2 mm	
SRM width	120 μm	
Silicon frame (outmost)	6 mm (w) × 6 mm (l)	
**Mechanical**		
CTE of Al	23.1 × 10^−6^ [K^−1^] ^g^	
CTE of Si_x_N_y_	2.3 × 10^−6^ [K^−1^] ^g^	
CTE of SiO_2_	0.55 × 10^−6^ [K^−1^] ^g^	
Thermal Conductivity of Al	237 [W/(K·m)] ^g^	
Thermal Conductivity of Si_x_N_y_	20 [W/(K·m)] ^g^	
Thermal Conductivity of SiO_2_	1.38 [W/(K·m)] ^g^	
Residual stress of Al	200 [MPa]	[26,27]
Residual stress of Si_x_N_y_	100 [MPa]	[28,29]
Elastic Modulus of Al	70 [GPa] ^g^	
Elastic Modulus of Si_x_N_y_	200 [GPa]	[28]
Poisson Ratio of Al	0.35 [a.u.] ^g^	
Poisson Ratio of Si_x_N_y_	0.23 [a.u.] ^g^	

^g^ Given from the FEM simulation tool (COMSOL Multiphysics^®^).

## Data Availability

The data presented in this study are available on request from the corresponding author. The data are not publicly available.

## Supplementary Materials

The following are available online at https://www.mdpi.com/article/10.3390/mi12070754/s1, Figure S1: Calculated film curvatures, Figure S2: Thickness of Al Joule heater and large-area mirror, Figure S3: Surface roughness at micron scale, Figure S4: Modal analysis at eigenfrequencies, Video S1: Reflected laser spot, Video S2: Electrothermal actuation of SRM-mirror.
