# Supplementary material for: Large-Area and Ultrathin MEMS Mirror Using Silicon Micro Rim
Source: Micromachines (Basel). 2021 Jun 26;12(7):754. doi: 10.3390/mi12070754 (PMC8308042; doi:10.3390/mi12070754)
Supplement: Supplementary file 1 [file micromachines-12-00754-s001.zip › micromachines-1265341-supplementary.pdf]

Supplementary Materials:

Communication

Large-area and Ultrathin MEMS Mirror using Silicon Micro Rim

Myeong-Su Ahn, Jaehun Jeon, Kyung-Won Jang and Ki-Hun Jeong*

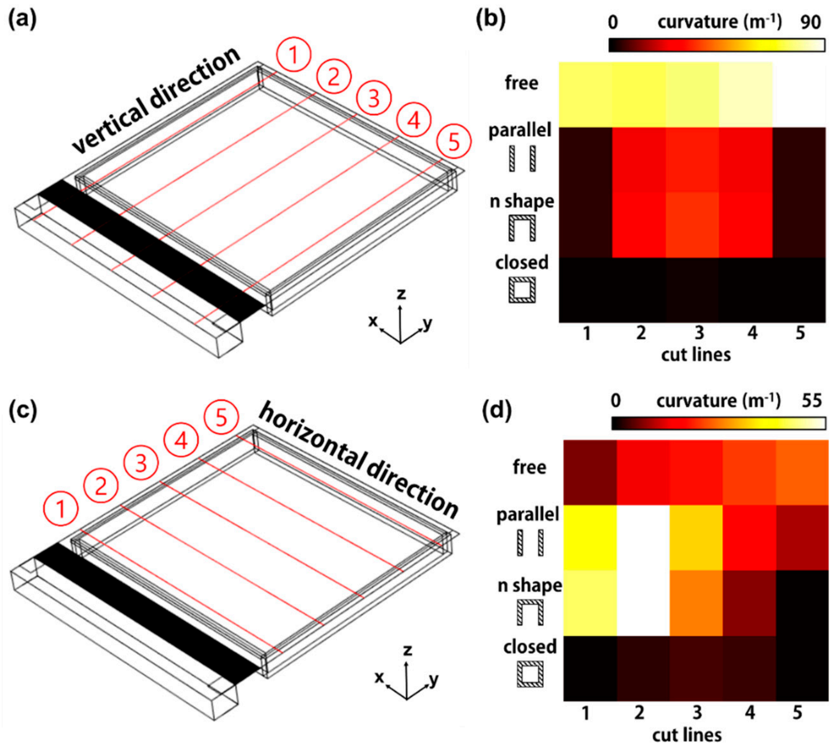

Figure S1. Calculated film curvatures of SRM-mirror depending on the shape of silicon rims such as parallel, one side-opened, closed shape and rim-free. Individual vertical lines (a) and corre-sponding curvatures (b). Individual horizontal lines (c) and corresponding curvatures (d).

Citation: Ahn, M.-S.; Jeon, J.; Jang, K.-W.; Jeong, K.-H. Large-Area and Ultrathin MEMS Mirror Using Silicon Micro Rim. *Micromachines* **2021**, *12*, 754. https://doi.org/10.3390/mi12070754

Academic Editor: Huikai Xie

Received: 2 June 2021
Accepted: 22 June 2021
Published: 26 June 2021

Publisher's Note: MDPI stays neutral with regard to jurisdictional claims in published maps and institutional affiliations.

Copyright: © 2021 by the authors. Submitted for possible open access publication under the terms and conditions of the Creative Commons Attribution (CC BY) license (http://creativecommons.org/licenses/by/4.0/).

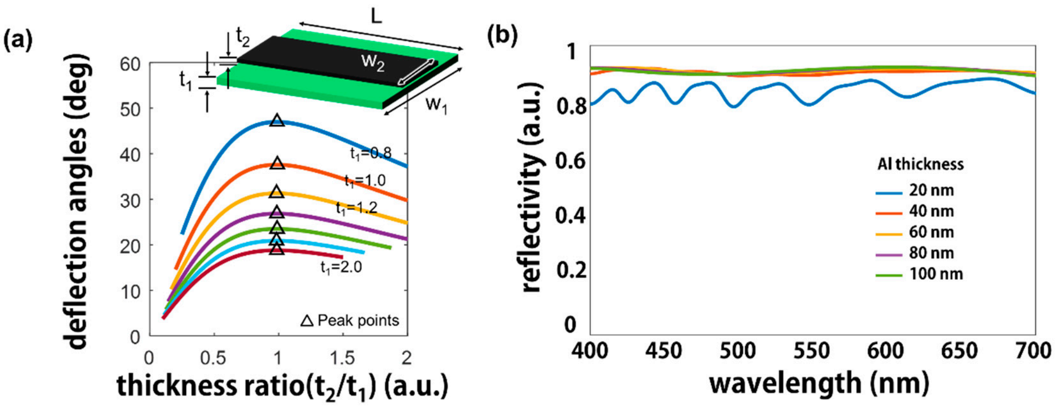

Figure S2. Thickness of Al Joule heater and large-area mirror. (a) Deflection angles of bimorph structures depending on the thicknesses of silicon nitride membrane and Al Joule heater. The bimorph structures show the largest deflection when the ratio of top layer (t_2) to bottom layer (t_1) is about 1. The thickness of silicon nitride was chosen to 1 um rather than 0.8 um, considering mechanical stability. (b) Reflectivity of Al film was calculated using three-dimensional finite difference time domain (FDTD) method. A 40 nm or thicker Al film shows high and uniform reflectivity over the visible range.

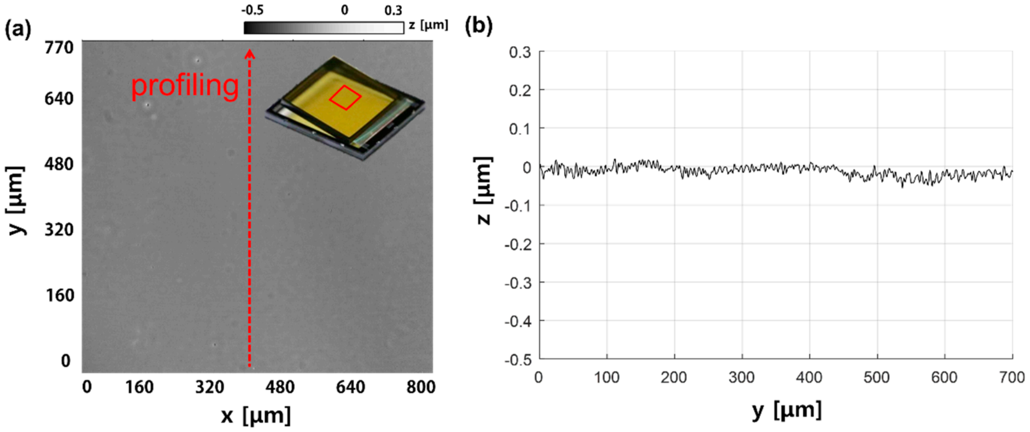

Figure S3. Surface roughness at micron scale. (a) Scanned raw data (2D) and (b) corresponding surface profile of the SRM-mirror, measured by using optical surface profiler (m-surf by Nanofocus AG).

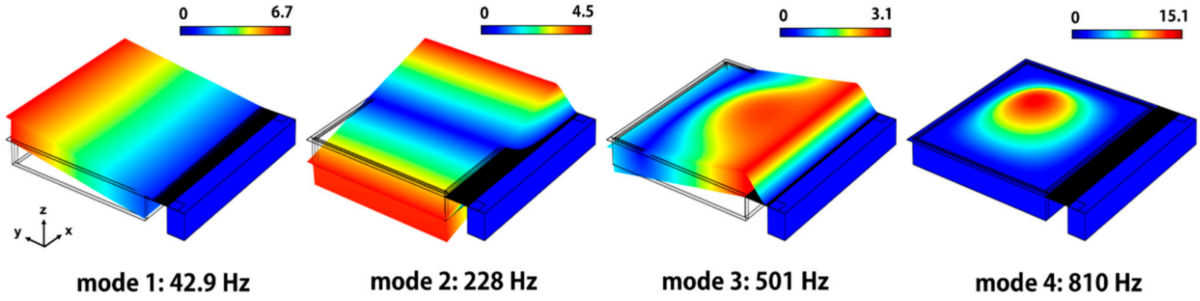

Figure S4. Modal analysis of the SRM-mirror. Diverse resonant actuation of the SRM-mirror depending on the eigenfre-quencies. The high-frequency modes including mode 2 ~ 4 exhibit undesirable actuations, which imply the SRM-mirror is not suitable for high-speed operation.

Video S1. Laser spot (633 nm) reflectred from the SRM-mirror during the elctrothermal actuation by applying sinusoidal AC voltages of 3 V_{PP} and 0.5 Hz.

Video S2. Electrothermal actuation of SRM-mirror by applying sinusoidal AC voltages of 3 V_{PP} and 0.5 Hz. Coherent light from 633 nm laser was incident at 60° and reflected at between 53° ~ 125°.
